# Demographics and outcomes of acute type A aortic dissection in young adults in southeastern China: impact of syndromic heritable thoracic aortic disease

**DOI:** 10.1080/07853890.2025.2457530

**Published:** 2025-01-28

**Authors:** Xin-fan Lin, Hang-qi Gao, Qing-song Wu, Yu-ling Xie, Liang-wan Chen, Lin-feng Xie

**Affiliations:** a Department of Cardiovascular Surgery, Fujian Medical University Union Hospital, Fuzhou, PR China; b Key Laboratory of Cardio-Thoracic Surgery (Fujian Medical University), Fujian Province University, Fuzhou, PR China; c Fujian Provincial Center for Cardiovascular Medicine, Fuzhou, PR China; d Department of Plastic Surgery and Regenerative Medicine, Fujian Medical University Union Hospital, Fuzhou, China

**Keywords:** Acute type A aortic dissection, young adult, heritable thoracic aortic disease, outcomes

## Abstract

**Background:**

This study aimed to investigate the demographics and to evaluate long-term outcomes of acute type A aortic dissection (ATAAD) in surgically treated patients ≤40 years in China.

**Methods:**

This study included patients aged ≤40 with ATAAD who underwent surgical treatment at our institution between 2015 and 2019. The patients were categorized into groups according to heritable thoracic aortic disease (HTAD) presence or absence. The primary outcomes were in-hospital and late mortality, and aortic-related re-intervention.

**Results:**

Our cohort included 141 patients aged ≤ 40, representing 14.6% of all patients with ATAAD treated during the same period. 35.5% (50 of 141) of these cases were associated with HTAD. Among them, only 16.0% were aware of their condition prior to the occurrence of ATAAD. Most patients underwent extensive arch procedures and in-hospital mortality rate of patients was 14.2%, which was higher in the HTAD group than in the non-HTAD group (20.0% vs. 11.0%, *p* = .142). The overall 7-year survival was 80.0%. Twenty patients required late aortic reoperations, with emergency surgeries accounting for 45% of the cases. The incidence of reoperation was significantly higher in the HTAD group than that in the non-HTAD group (*p* = .03). In addition, the late aortic reoperation remained a risk factor for long-term survival after adjusting for clinical factors.

**Conclusions:**

The prevalence of HTAD is high in the cohort of younger patients with ATAAD. HTAD is associated with high rates of in-hospital mortality and late reoperation. Extensive primary aortic repair is safe and has long-term benefits in young patients with ATAAD. Regular imaging assessment of the thoracoabdominal aorta after surgery is imperative for improving the long-term prognosis.

## Introduction

Acute type A aortic dissection (ATAAD) in young adults presents with distinct clinical features, including a higher incidence of heritable thoracic aortic disease (HTAD) and more extensive dissection involvement [1]. Despite timely surgical interventions, the risk of mortality remains significant, and some patients may require late aortic reinterventions [2, 3]. The HTAD refers to a spectrum of disorders associated with underlying familial components or pathogenic genetic variants, frequently leading to aortic root and ascending aorta aneurysms or dissections [4]. They have been divided into syndromic (Marfan, Loeys–Dietz and vascular Ehlers–Danlos syndromes) and nonsyndromic entities, with the latter lacking extracardiovascular involvement. ATAAD is relatively rare in young individuals, with a reported incidence of 1.8–2.5% [5, 6]. In China, the mean age of patients with ATAAD (51.8 years) is nearly a decade younger than the figures reported in the International Registry of Acute Aortic Dissection (IRAD) database [7, 8]. Given the lack of sufficient research on this population, and the long-term impact of aortic disease on the quality of life and life expectancy in young patients, we aim to analyse the influence of HTAD on the prognosis of young patients (≤40 years) with ATAAD. Specifically, we compare the demographics, early outcomes and long-term outcomes between HTAD and non-HTAD patients.

## Methods

### Study population

Patients diagnosed and treated for ATAAD at our institution between 2015 and 2019 were enrolled in this retrospective analysis. The study cohort included all adult patients aged ≤ 40 years, with symptom onset within 14 days. The clinical diagnostic criteria for HTAD were based on intraoperative findings of abnormal aortic wall (such as thinning and stiffness, excluding acquired aortopathy), with or without typical syndromic features, such as skeletal abnormalities, ectopia lentis and a positive family history. The genetic testing was used as auxiliary methods to improve the diagnosis and was recommended on patients clinically diagnosed with HTAD or those with a positive family history. A diagnosis of HTAD can be made based on either positive clinical findings or positive genetic testing (or both).

### Procedure

The primary objective of surgery is to resect the primary entry tear. The ATAAD repair strategy is more aggressive in younger patients at our institution. Root treatment was performed to prevent acute complications, such as dissection and rupture. Patients underwent various root procedures depending on the degree of dilatation and the presence of structurally diseased valves. Patients with HTAD might more often receive root replacement rather than repair. Surgical intervention is necessary if the dissection involves the arch. Our routine arch procedure is hemiarch replacement combined with triple-branched stent graft (TBSG) implantation. However, if the most proximal entry tear was located in the arch, a total arch replacement was performed using a four-branch prosthetic graft. Axillary and femoral artery cannulation was routinely used for cardiopulmonary bypass. Femoral artery cannulation was considered for patients undergoing only root procedures. Axillary artery cannulation was considered for patients undergoing only distal procedures. All operations were performed by the same experienced surgeon. Survivors underwent regular assessments through clinical visits and computed tomography angiography at five specific time points (before discharge and at 3, 6, 12 and 24 months postoperatively). Telephone interviews took place semiannually. Late reoperation included reintervention of the proximal and distal aorta. The possible causes included residual dissection or graft-related complications.

### Statistical analysis

Statistical analysis was conducted using R software version 4.3.2 (R Foundation for Statistical Computing, Vienna, Austria). Continuous variables are presented as the mean ± standard deviation or median (interquartile range). Categorical variables are represented as counts and proportions. Comparisons between the groups were performed using Chi-squared tests for categorical variables and *t*-tests for continuous variables. Missing values were imputed using the most commonly represented values for categorical variables or the median values for continuous variables. The Kaplan–Meier (K–M) method was employed to calculate the overall survival and reoperation rates, which were then compared using the log-rank test. Univariate and multivariate logistic regression and Cox proportional hazards models were used to evaluate the factors associated with early- and long-term outcomes. Variables including demographic characteristics, preoperative status, dissection-related imaging findings and intraoperative data, those with *p* ≤ .20 in the univariate regression analysis, were selected as potential candidates for multivariate analysis. Significance was determined as a two-tailed *p* ≤ .05.

## Results

### Demography

**Figure 1.**
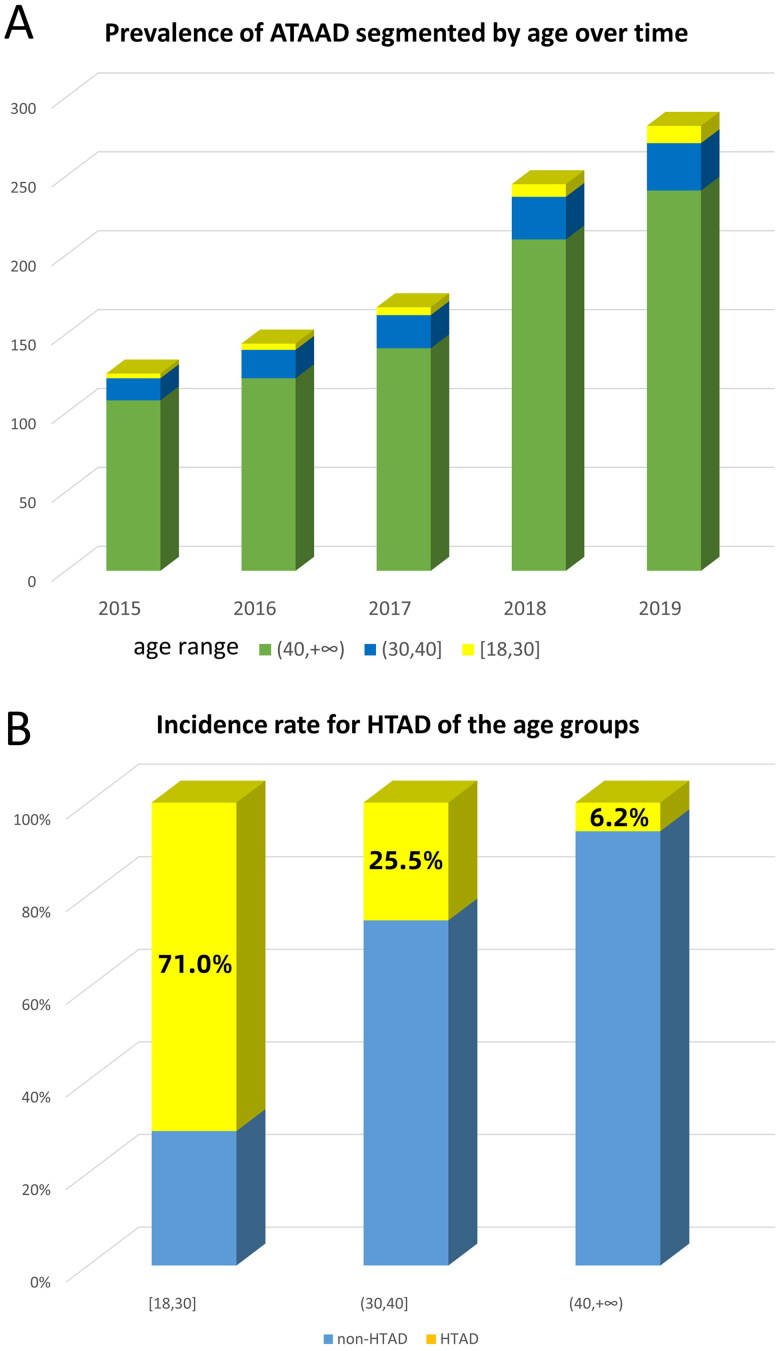
(A) Prevalence of ATAAD segmented by age over time. (B) Incidence rate for HTAD of the age groups.

From January 2015 to December 2019, a total of 963 patients underwent surgical treatment for ATAAD in our institution. The prevalence of ATAAD showed a gradually increasing trend (Figure 1(A)). There was a noticeable variation in the prevalence of HTAD among the different age groups (71.0% in ages 18–30 vs. 25.5% in patients aged 30–40 vs. 6.2% in patients aged >40) (Figure 1(B)). The study cohort consisted of 141 patients ≤40 years (14.6%) with ATAAD. The mean age was 33.6 ± 4.7 years, and the vast majority were male (75.2%). Fifty patients were diagnosed with HTAD, with only 8 of them having been previously diagnosed. Marfan syndrome (MFS) had the highest prevalence (Table 1). Within the HTAD group, the patients were younger (*p* < .001), had a lower body mass index (*p* < .001), and were more likely to have a family history of aortic disease (*p* = .001). In contrast, aortic dissection was significantly associated with hypertension (*p* < .001) in the non-HTAD group.

**Table 1.** Baseline characteristics.

Parameters	All patients (*n* = 141)	HTAD group (*n* = 50)	Non-HTAD group (*n* = 91)	*p* Value
Age	33.6 ± 4.7	30.5 ± 5.2	35.4 ± 3.4	<.001
Male	106 (75.2)	39 (78.0)	66 (72.5)	.565
BMI	23.9 ± 4.0	20.8 ± 3.2	25.6 ± 3.5	<.001
Aortopathy	50 (35.5)	50 (100)	–	N/A
MFS	44 (31.2)	44 (88.0)	–	N/A
Loeys–Dietz syndrome	4 (2.8)	4 (8.0)	–	N/A
Vascular Ehlers–Danlos syndrome	2 (1.4)	2 (4.0)	–	N/A
Pregnancy	4 (2.8)	4 (8.0)	0	.006
Positive family history	21 (14.9)	14 (28.0)	7 (7.7)	.001
Aortic surgery history	5 (3.5)	0	5 (5.5)	.091
Hypertension	58 (41.1)	2 (4.0)	56 (61.5)	<.001
Time from onset to arrivals (h)	5.0 (3.0,7.0)	5.0 (3.0,8.0)	4.0 (2.0,6.0)	.056
BAV	5 (3.5)	0	5 (5.5)	.091
LVEF	61.6 ± 9.1	61.0 ± 9.9	62.0 ± 8.7	.535
Aortic insufficiency	76 (53.9)	39 (78.0)	37 (40.7)	<.001
Moderate	55 (39.0)	24 (48.0)	31 (34.1)	.105
Severe	21 (14.9)	15 (30.0)	6 (6.6)	<.001
Root diameter	46.3 ± 10.8	52.7 ± 13.1	42.7 ± 7.3	<.001
Proximal entry tear location				.192
Root	8 (5.7)	2 (4.0)	6 (6.6)	
Ascending	103 (73.0)	34 (68.0)	69 (75.8)	
Arch	20 (14.2)	6 (12.0)	14 (15.4)	
No found	10 (7.1)	8 (16.0)	2 (2.2)	
Extension of dissection				
Supra-aortic vessels	91 (64.5)	32 (64.0)	59 (64.8)	.921
Abdominal Aorta	102 (72.3)	33 (66.0)	69 (75.8)	.212
Iliac vessels	48 (34.0)	11 (22.0)	37 (40.7)	.025
Preoperative malperfusion				
Coronary	19 (13.5)	10 (20.0)	9 (9.9)	.093
Cerebral	12 (8.5)	3 (6.0)	9(9.9)	.428
Spinal	4 (2.8)	0	4 (4.4)	.132
Mesenteric	8 (5.7)	5 (10.0)	3 (3.3)	.091
Renal	22 (15.6)	11 (22.0)	11 (12.1)	.121
Peripheral	3 (2.1)	0	3 (3.3)	.193
GERAADA score (%)	12.9 ± 6.2	12.8 ± 7.0	13.0 ± 5.9	.857

BMI: body mass index; BAV: bicuspid aortic valve; GERAADA: German Registry of Acute Aortic Dissection Type A; HTAD: heritable thoracic aortic disease; LVEF: left ventricular ejection fraction; MFS: Marfan syndrome.

Values are given as median and interquartile range or numbers and percentages.

### Dissection-related data

HTAD patients showed significant dilation of the roots (*p* < .001). 30% of the patients (n = 15) exhibited severe aortic regurgitation. There was a higher prevalence of iliac vessel involvement in non-HTAD patients (*p* = .025). The ascending aorta was the most common entry site (73%). The entry site was located in the aortic arch in 20 patients (14.2%).

### Intraoperative data

More patients in the HTAD group underwent a root procedure (*p* < .001), with the Bentall procedure being the most common (84.0%). In the non-HTAD group, 41.8% of patients underwent the patch neointima technique (PNT). Most patients (79.4%) underwent an arch procedure. The arch reconstruction included two main techniques, with the TBSG accounting for 63.1% and total arch replacement with frozen elephant trunk (TAR + FET) for 16.3% (Table 2). In all cases, the preferred cannulation sites were the femoral and axillary arteries. More patients in the HTAD group underwent single femoral or axillary artery cannulation (*p* = .013).

**Table 2.** Intraoperative data.

Parameters	All patients (*n* = 141)	HTAD group (*n* = 50)	Non-HTAD group (*n* = 91)	*p* Value
Root procedure	106 (75.2)	46 (92.0)	60 (65.9)	<.001
David	6 (4.3)	4 (8.0)	2 (2.2)	
Bentall	62 (44.0)	42 (84.0)	20 (22.0)	
PNT	38 (27.0)	0	38 (41.8)	
Arch procedure	112 (79.4)	36 (72.0)	76 (83.5)	.106
TAR + FET	23 (16.3)	9 (18.0)	14 (15.4)	.688
HAR + TBSG	89 (63.1)	27 (54.0)	62 (68.1)	.091
HAR	22 (15.6)	8 (16.0)	14 (15.4)	.923
CABG	7 (5.0)	7 (14.0)	0	<.001
DHCA	101 (71.6)	33 (66.0)	68 (74.7)	.271
Cerebral perfusion	112 (79.4)	36 (72.0)	76 (83.5)	.195
Antegrade	103 (73.0)	32 (64.0)	71 (78.0)	
Retrograde	9 (6.4)	4 (8.0)	5 (5.5)	
Arterial cannulation				.013
Axillary and femoral	111 (78.7)	30 (60.0)	81 (89.0)	
Axillary alone	8 (5.7)	4 (8.0)	4 (4.4)	
Femoral alone	22 (15.6)	16 (32.0)	6 (6.6)	

CABG: coronary artery bypass grafting; DHCA: deep hypothermic circulatory arrest; HAR: hemiarch replacement; HTAD: heritable thoracic aortic disease; PNT: patch neointima technique; TAR + FET: total arch replacement + frozen elephant trunk; TBSG: triple-branched stent graft.

### Clinical outcomes

**Figure 2.**
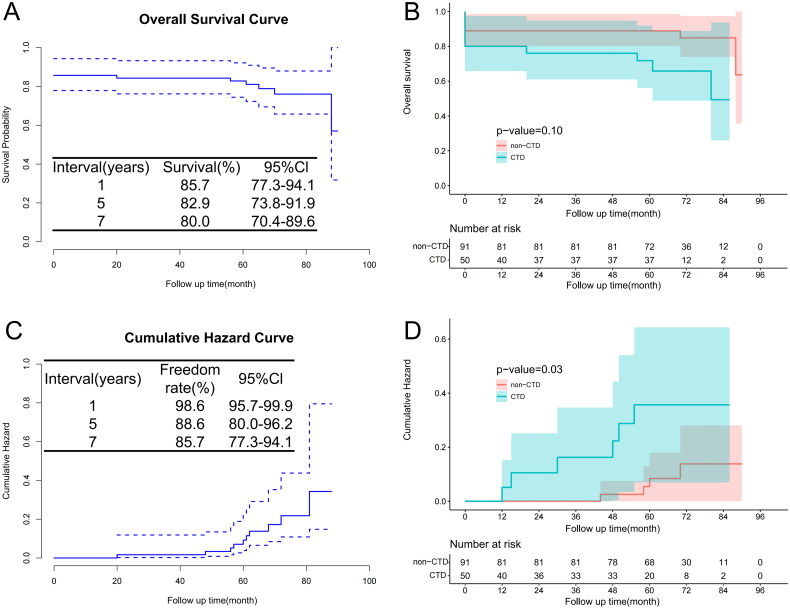
Kaplan–Meier’s analysis of young patients with ATAAD. (A) Overall survival for the entire cohort; (B) overall survival in patient with HTAD and without HTAD; (C) cumulative aortic reoperation for the entire cohort; (D) cumulative aortic reoperation in patient with HTAD and without HTAD.

The in-hospital mortality rate was 14.2% in patients aged ≤40 (20.0% in the HTAD group vs. 11.0% in the non-HTAD group, *p* = .142; 9.1% in the entire cohort). In the HTAD groups, the primary cause of death was rupture of the residual dissection (6 of 10). Thirteen deaths were observed during a mean follow-up of 68.00 (62.00–77.00) months (8 in the HTAD group and 5 in the non-HTAD group). Among them, eight (61.5%) had undergone late aortic reoperation. The 1-, 5- and 7-year survival rates were 80.0% [95% confidence interval (CI), 63.1–96.9%], 72.0% (95% CI, 53.1–90.9%) and 68.0% (95% CI, 48.3–87.7%) in the HTAD group, and 88.9% (95% CI, 79.3–98.4%), 88.9% (95% CI, 79.3–98.4%) and 86.7% (95% CI, 76.3–97.0%) in the non-HTAD group, respectively. K–M analysis showed no significant difference in overall survival between the groups (Figure 2(A,B)). Patients in the HTAD group exhibited a higher incidence of peripheral vascular complications, specifically, subclavian artery occlusion and internal iliac artery aneurysms (Supplementary Material, Table S1).

### Reoperations data

Aortic reoperation was performed in 20 patients, 45% of whom underwent emergency surgery, with a mortality rate of 25% (Table 3). The freedom from reoperation in HTAD group at 1-, 5- and 7 years were 96.0% (95% CI, 87.7–98.6%), 76.0% (95% CI, 58.0–94.0%) and 76.0% (95% CI, 58.0–94.0%), respectively. The non-HTAD group had rates of 100%, 95.6% (95% CI, 89.3–98.2%) and 91.1% (95% CI, 82.5–97.5%), respectively. K–M analysis revealed that the HTAD group had significantly higher rates of aortic reoperation (*p* = .03) (Figure 2(C,D)).

**Table 3.** Reoperation data.

Case	HTAD or not	First surgery			Reoperation			
		Age	Root	Arch and distal	Years after surgery	Indication	Reoperation	status
1	Yes	30	Bentall	–	2.3	Arch dissection	Bentall + TAR	In-hospital death
2	Yes	27	Bentall	–	1.0	Arch dissection	TAR	Survival (3.8 years)
3	Yes	35	Bentall	TAR + FET	4.2	Anastomotic leakage	TAR	In-hospital death
4	Yes	31	Bentall	TBSG	0.9	Endoleak	Bentall + TAR	Death (1.0 years)
5	Yes	32	Bentall	HAR	4.0	Left subclavian artery occlusion	CSB	Death (2.3 years)
6	Yes	35	Bentall	TBSG	2.1	Left subclavian artery occlusion	CSB	Survival (4.0 years)
7	Yes	29	Bentall	TBSG	5.0	Thrombosis of mechanical valve	Bentall	In-hospital death
8	Yes	22	David	–	2.5	Severe AI	Bentall	Death (5.0 years)
9	Yes	33	–	TBSG	2.4	Severe AI	AVR	Survival (4.8 years)
10	Yes	39	Bentall	TBSG	1.3	Distal dissection	TEVER	Survival (3.5 years)
11	Yes	33	Bentall	TAR	3.3	Distal aneurysm	TEVER	Survival (3.1 years)
12	Yes	33	Bentall	–	2.7	Distal aneurysm	TEVER	Survival (5.2 years)
13	No	31	Bentall	TBSG	1.1	Endoleak	Bentall + TAR	In-hospital death
14	No	31	PNT	TAR + FET	1.0	Root aneurysm	Bentall	In-hospital death
15	No	22	–	TBSG	3.5	Root aneurysm	Bentall	Survival (4.8 years)
16	No	37	PNT	TBSG	6.3	Severe AI	AVR	Survival (1.2 years)
17	No	29	Bentall	TBSG	3.7	Distal dissection	TEVER	Survival (2.5 years)
18	No	35	–	TAR + FET	4.8	Distal dissection	TEVER	Survival (2.0 years)
19	No	34	PNT	TBSG	2.0	Distal dissection	TEVER	Survival (3.6 years)
20	No	39	Bentall	–	4.1	Distal aneurysm	TEVER	Survival (2.0 years)

AI: aortic insufficiency; AVR: aortic valve replacement; CSB: carotid‐subclavian bypass; HAR: hemiarch replacement; HTAD: heritable thoracic aortic disease; PNT: patch neointima technique; TAR + FET: total arch replacement + frozen elephant trunk; TBSG: triple-branched stent graft; TEVER: thoracic endovascular aortic repair.

### Logistic regression

The German Registry of Acute Aortic Dissection Type A score (odds ratio (OR) 1.15; *p* = .02), left ventricular ejection fraction (OR 0.90; *p* = .013) and preoperative cerebral malperfusion (OR 8.68; *p* = .041) were significantly associated with in-hospital mortality (Table 4). Late aortic reoperation (hazard ratio (HR), 6.58; *p* = .001) and aortic insufficiency (AI) (HR, 3.94; *p* = .033) were independent risk factors for long-term mortality. Furthermore, after adjusting for supra-aortic dissection and proximal entry tear location in the aortic arch, which were significant factors in the univariate analysis, HTAD was an independent predictor of late reoperation (HR, 4.98; *p* = .031). Further analysis shows that in patients with HTAD, smoking (HR, 3.66; *p* = .045), supra-aortic dissection (HR, 7.10; *p* = .012) and proximal entry tear location in the arch (HR, 3.89; *p* = .028) are risk factors for aortic reoperation.

**Table 4.** Risk factors for outcomes.

Factors associated with	HR/OR (95% CI)	*p* Value
In-hospital mortality	OR	
HTAD	3.97 (0.66–23.79)	.131
GERAADA score	1.15 (1.02–1.30)	.020
LVEF	0.90 (0.83–0.98)	.013
Cerebral malperfusion	8.68 (1.09–68.60)	.041
Long-term mortality	HR	
HTAD	1.62 (0.54–4.81)	.387
Aortic reoperation	6.58 (2.07–20.90)	.001
AI	3.94 (1.12–13.87)	.033
Aortic reoperation	HR	
HTAD	4.98 (1.16–21.28)	.031
Supra-aortic dissection	8.90 (1.16–40.00)	.016
Proximal entry tear location in arch	4.24 (1.00–17.86)	.049
Aortic reoperation for patients with HTAD	HR	
Smoking	3.66 (1.03–13.01)	.045
Supra-aortic dissection	7.10 (1.53–33.00)	.012
Proximal entry tear location in arch	3.89 (1.16–13.00)	.028

AI: aortic insufficiency; GERAADA: German Registry of Acute Aortic Dissection Type A; HTAD: heritable thoracic aortic disease; HR: hazard ratio; LVEF: left ventricular ejection fraction; OR: odds ratio.

## Discussion

ATAAD commonly onsets during the sixth decade of life. In the USA [9], a proportion of 8.7% was reported in patients aged < 40 years, whereas in our cohort, the proportion was high at 14.6%. Hypertension is the most common risk factor for ATAAD. In China, the prevalence of hypertension has increased over the years, with rates ranging from 11.3% in 1991 [10] to 26.6% in 2011 [11]. Meanwhile, the age of onset has been decreasing. A recent study reported that 26% of young Chinese individuals are affected by hypertension, with 87% not receiving any treatment and 73% being unaware of their condition [12]. The high prevalence of hypertension and poor medication adherence could contribute to the rising incidence of ATAAD among young people in China. The bicuspid aortic valve (BAV) is also associated with an increased risk of ATAAD at a much younger age. This may be associated with persistent aortic dilation and abnormal haemodynamics in BAV. Although most cases are sporadic, the prevalence of BAV in first-degree relatives is approximately 6% [13]. However, the incidence of BAV is markedly lower in China than that in Western countries.

The high prevalence of HTAD (35.5%) is the most distinctive feature of this rare patient population. The high risk of life-threatening adverse aortic events in HTAD patients at a young age is due to the rapid enlargement of the aortic root caused by connective tissue deformation. MFS is the most common genetic disorder of the connective tissues in China, with an incidence of 10.2 per 100,000 individuals [14]. Owing to a lack of awareness regarding HTAD among the general public, many patients do not realize that they have the disease until ATAAD occurs. This could explain the high proportion of patients with HTAD in our cohort. The current guidelines recommend screening for first-degree relatives of individuals with HTAD [15]. A family history of MFS was detected in 49% of individuals [15]. Once diagnosed, regular aortic imaging is crucial for managing the risk of acute aortic dissection. Elective composite valve graft procedures and valve-sparing aortic root replacement procedures have a low operative mortality, with favourable long-term prognosis and high quality of life [16]. The surgical thresholds for prophylactic aortic root replacement vary depending on the specific disease. For MFS, the threshold is 50 mm; for Loeys–Dietz syndrome, it is 45 mm; and for nonsyndromic HTAD, the threshold is also considered to be 45 mm [17]. Interestingly, a positive family history was also observed in the patients without HTAD. A Denmark study reported that the first-degree relatives of individuals with aortic aneurysms and dissection had an elevated risk of the same conditions, even after adjusting for HTAD [18]. Conventional cardiovascular risk factors do not account for familial clustering.

**Figure 3.**
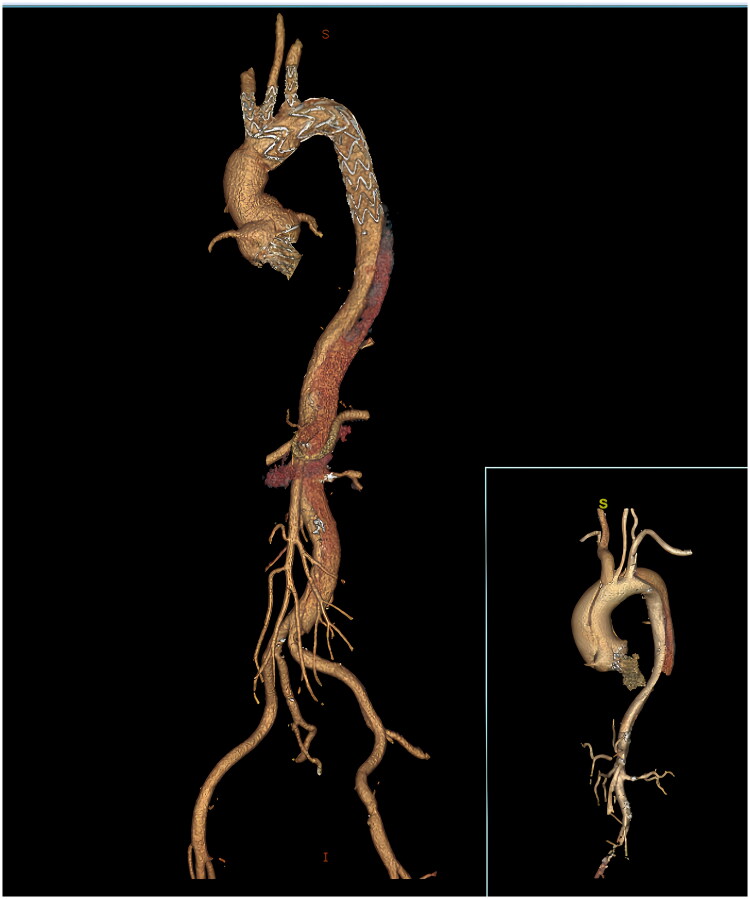
The hemiarch replacement combined with triple-branched stent graft.

Studies have demonstrated the efficacy of valve-sparing root replacement (VSRR) as a durable technique that eliminates the need for anticoagulation [19]. This issue is significant, especially for patients with an extended life expectancy. However, whether VSRR is feasible in patients with HTAD remains controversial. As noted by Ram et al. [20], the rationale for performing this procedure stems from the absence of intrinsic valve pathology, with functional aortic regurgitation linked to root dilatation in these patients. The advantages of preserving native valve tissue may outweigh the potential risks of recurrent AI in young HTAD patients [21]. We conducted modified VSRR-PNT procedures [22] in patients with ATAAD without HTAD. This technique involves the utilization of three teardrop-shaped patches to bolster the sinus segment and preserve the geometric integrity of the aortic root. However, the enlargement and irregular configuration of the aortic root in patients with HTAD, coupled with fragile tissue characteristics, pose challenges when conducting repairs using polyester surgical patches within constrained timeframes. ATAAD in young individuals commonly extends to the descending aorta. Typically, TAR + FET is recommended when the aortic arch is involved. To promptly restore perfusion to vital organs, many patients undergo TBSG placement at our institution (Figure 3). This technique eliminates the need for anastomosis of the descending aorta and supra-aortic arteries and has been proven safe and effective [23]. Endovascular stent graft placement alone has shown suboptimal long-term results in patients with HTAD and ATAAD, as continuous compression of the aortic wall in the graft landing zones can lead to future dilation and endoleaks. Therefore, it is typically used as a bail out procedure or temporary measure before definitive open surgery. In patients with HTAD and ATAAD, open stent graft placement not only simplifies arch procedures, but also remodels the distal aorta, ultimately expanding and stabilizing the true lumen. Furthermore, in cases where distal reintervention is deemed necessary, the graft can serve as a suitable landing zone, thereby enhancing the safety of late-stage thoracoabdominal aortic replacement.

The physical condition of young patients allows them to tolerate more extensive primary repairs, resulting in better early- and long-term survival [24]. However, for patients with HTAD, the pathological condition of the aorta is persisting, and there remains a higher likelihood of reintervention. In our cohort, the mortality rate of the reinterventions is relatively high, primarily due to the significant proportion (45%) of surgeries performed for acute aortic events. This emphasizes the importance of regular imaging assessment of the thoracoabdominal aorta. We acknowledge the in-hospital mortality rates were high (14.2%) in our cohort. But we do not believe aggressive surgical strategies are the primary reason for increased mortality rate. Instead, the high incidence of distal aortic rupture caused by connective tissue disorders, as well as the higher proportion of impoverished patients compared to other cohorts (eight patients chose to discontinue treatment due to the financial burden caused by prolonged mechanical ventilation dependence), has led to this result.

Although ATAAD is rare in young populations, its prognosis is currently unsatisfactory. It presents significant challenges for cardiovascular surgeons in terms of both surgical treatment and post-discharge health management. This study, based on clinical data from the largest aorta centre in southeastern China, provides an in-depth analysis of the characteristics, surgical management and long-term outcomes of young patients (≤40 years) with ATAAD, with a particular focus on those with HTAD. The findings offer valuable practical experience and data support for the diagnosis and treatment of young ATAAD patients, especially those with HTAD, and provide important references for optimizing treatment strategies.

## Limitations

As a single-centre retrospective study with small sample size, its external validity could be limited. The fact that genetic testing was not routinely conducted is an issue that cannot be ignored. Apart from patients clinically diagnosed with HTAD, we only recommend genetic testing for those with a positive family history. This may lead to missed diagnoses of mild phenotypic features of aortopathies. Although the overall follow-up rate of 95.9% was satisfactory, the follow-up duration may not have been long enough for younger patients. It is imperative that future studies include multi-centre studies with large sample sizes and extended follow-up duration.

## Conclusions

The prevalence of HTAD is high in the cohort of younger patients with ATAAD. HTAD is associated with high rates of in-hospital mortality and late reoperation. Extensive primary aortic repair is safe and has long-term benefits in young patients with ATAAD. Regular imaging assessment of the thoracoabdominal aorta after surgery is imperative for improving the long-term prognosis.

## Supplementary Material

Table S1.docx

## Data Availability

The data that support the findings of this study are available on request from the corresponding author.
