# Supplementary material for: Demographics and outcomes of acute type A aortic dissection in young adults in southeastern China: impact of syndromic heritable thoracic aortic disease
Source: Ann Med. 2025 Jan 28;57(1):2457530. doi: 10.1080/07853890.2025.2457530 (PMC11776063; doi:10.1080/07853890.2025.2457530)
Supplement: Table S1.docx [file IANN_A_2457530_SM0843.docx]

| **Table S1.** Short and long-term events | | | | | |
| --- | --- | --- | --- | --- | --- |
| **Parameters** | **All patients**  **(n= 141)** | | **HTAD Group**  **(n= 50)** | **Non- HTAD Group**  **(n= 91)** | ***p*-**  **Value** |
| **Early events** |  | |  |  |  |
| In-hospital mortality | 20 (14.2) | | 10 (20.0) | 10 (11.0) | .142 |
| residual dissection rupture | 7 (5.0) | | 5 (10.0) | 2 (2.2) |  |
| multiple organ failure  LCOS  septic shock  cerebrovascular accident | 6 (4.3)  4 (2.8)  2 (1.4)  1 (0.7) | | 3 (6.0)  2 (4.0)  0  0 | 3 (3.3)  2 (2.2)  2 (2.2)  1 (1.1) |  |
| ICU length of stay | 4.6±6.3 | | 3.7±5.1 | 5.2±6.8 | .349 |
| Respiratory insufficiency |  | |  |  |  |
| Prolonged ventilation(>48h) | 56 (39.7) | | 13 (26.0) | 43 (47.3) | .014 |
| Tracheotomy | 7 (5.0) | | 1 (2.0) | 6 (6.6) | .230 |
| Volume on the first 24h | 444.1±264.4 | | 424.4±258.4 | 455.0±269.9 | .646 |
| Re-exploration for bleeding | 4 (2.8) | | 2 (4.0) | 2 (2.2) | .537 |
| CRRT | 12 (8.5) | | 4 (8.0) | 8 (8.8) | .872 |
| Paraplegia | 5 (3.5) | | 2 (4.0) | 3 (3.3) | .829 |
| Stroke | 14 (9.9) | | 4 (8.0) | 10 (10.9) | .570 |
| LCOS | 12 (8.5) | | 5 (10.0) | 7 (7.7) | .639 |
| **Long-term events** |  | |  |  |  |
| Mean follow-up (months) | 70.5±1.4 | | 68.5±2.6 | 71.4±1.6 |  |
| Overall survival | 108 (76.6) | | 32 (64.0) | 76 (83.5) | .008 |
| Reoperations | 20 (14.2) | | 12 (24.0) | 8 (8.8) | .013 |
| Stroke | 3 (2.1) | | 1 (2.0) | 2 (2.2) | .938 |
| Bleeding | 1 (0.7) | | 1 (2.0) | 0 | .176 |
| Endocarditis | 1 (0.7) | | 0 | 1 (1.1) | .457 |
| New on-set moderate/severe AI | 6 (4.3) | | 2 (4.0) | 4 (4.4) | .911 |
| Mechanical aortic valve thrombosis | | 1 (0.7) | 1 (2.0) | 0 | .176 |
| Root diameter | | 27.2±2.2 | 26.4±2.3 | 26.6±2.2 | .612 |
| Subclavian artery occlusion  (Left or right) | | 12 (8.5) | 8 (16.0) | 4 (4.4) | .018 |
| Internal iliac artery aneurysm  (Left or right) | | 7 (5.0) | 5 (10.0) | 2 (2.2) | .041 |

**Values are given as median and interquartile range or numbers and percentages.**

**AI**, aortic insufficiency; **CRRT**, continuous renal replacement therapy; **LCOS**, low cardiac output syndrome.
